# Association Between Hyperchloremia and Neurological Outcomes in Traumatic Brain Injury: A Narrative Review

**DOI:** 10.3390/healthcare14050696

**Published:** 2026-03-09

**Authors:** Philippa McIlroy, Mahesh Ramanan, Kyle C. White, Kevin B. Laupland, Mark J. Hackett, Gaewyn Ellison, Robert McNamara

**Affiliations:** 1 Cairns Hospital, Cairns, QLD 4870, Australia; philippa.mcilroy@health.qld.gov.au; 2 The Prince Charles Hospital, Chermside, QLD 4032, Australia; 3 Royal Brisbane and Women’s Hospital, Herston, QLD 4029, Australia; 4 The George Institute for Global Health, University of New South Wales, Kensington, NSW 2033, Australia; 5 School of Medicine, Queensland University of Technology, Kelvin Grove, QLD 4059, Australia; 6 Princess Alexandra Hospital, Woolloongabba, QLD 4102, Australia; 7 School of Medicine, The University of Queensland, St Lucia, QLD 4072, Australia; 8 School of Molecular and Life Sciences, Curtin University, Bentley, WA 6102, Australia; mark.j.hackett@curtin.edu.au (M.J.H.);; 9 Royal Perth Hospital, Perth, WA 6000, Australia; robert.mcnamara@health.wa.gov.au

**Keywords:** traumatic brain injury, chloride, intracranial pressure, neurological outcome, mortality

## Abstract

**Background/Objectives**: Traumatic brain injury (TBI) is a leading cause of morbidity and mortality worldwide. Electrolyte disturbances are common in this patient cohort, with serum chloride frequently elevated. Chloride dysregulation may be associated with poor neurological outcomes through mechanisms including paradoxical gamma amino butyric acid receptor excitation, cytotoxic edema, and ferroptosis. The aim of this review was to evaluate the relationship between serum chloride levels and outcomes in patients with TBI. **Methods**: A literature review was performed to identify all potential studies that reported on serum chloride levels and TBI. All study types and patient groups were included. Studies were included if they reported on serum chloride measurements as well as outcomes such as mortality, surgical intervention, intracranial pressure, and neurological/functional outcome scores in patients with TBI. References and citations were also reviewed. **Results**: A small number of mostly retrospective studies with modest patient numbers demonstrate an association between high chloride levels and increased mortality in patients with TBI, with this relationship persisting independent of hypernatremia. Recent large, randomized trials showed that balanced crystalloid solutions, despite lower chloride content, may be associated with worse outcomes in TBI patients compared to saline. No studies directly correlated chloride levels with intracranial pressure measurements. Chloride level rather than total chloride load appears more strongly associated with adverse outcomes, with non-hypertonic saline sources contributing substantially to chloride burden. Mechanistic evidence links chloride channel dysregulation to ferroptosis and cytotoxic edema, with sex-specific patterns of transporter expression. **Conclusions**: Limited available evidence suggests that hyperchloremia is independently associated with increased mortality in TBI though causality remains unestablished. The findings regarding balanced solutions challenge conventional fluid management assumptions and highlight the complexity of chloride’s role in TBI pathophysiology. The absence of studies directly correlating chloride with intracranial pressure represents a critical evidence gap. Future studies with larger patient numbers, prospective designs, and multimodal neuromonitoring should further define these relationships to inform evidence-based chloride management strategies.

## 1. Introduction

Traumatic brain injury (TBI) is a leading cause of morbidity and mortality worldwide [1]. Among the various physiological disturbances observed in TBI patients, electrolyte imbalances—particularly involving chloride—have gained increasing attention. Chloride imbalance is common in the general ICU population, with 56% of ICU patients found to have abnormal chloride levels on admission, and over 70% found to have hyperchloremia by day 4 of ICU admission [2].

Chloride (Cl^−^), the principal extracellular anion, is vital in maintaining acid–base balance, osmolality, and fluid distribution [3]. In neuronal cells it is crucial for synaptic transmission, mediated by the inhibitory neurotransmitters gamma-aminobutyric acid (GABA) and glycine [4]. When these inhibitory neurotransmitters bind to their receptors, it alters the cell membrane permeability to chloride ions, resulting in a net shift in chloride across the neuronal membrane. This leads to hyperpolarization of the cell membrane, and subsequent neuronal inhibition. The concentration of intracellular chloride determines both the magnitude and polarity of GABA-mediated neurotransmission [4].

Chloride also plays an important role in the regulation of intracellular volume. Neurons lack rigid cell walls and are unable to tolerate large pressure fluctuations between intracellular and extracellular compartments without significant volume shifts [5]. Neurons lack aquaporins, thus the movement of water largely accompanies the movement of solutes across the cell membrane. The intracellular volume is thus tightly regulated by the electrochemical balance between inward and outward flux of chloride and other ions through cation- chloride cotransporters (CCCs) and other channels.

In disease states, serum chloride levels may be altered by autoregulatory mechanisms or in the case of TBI, therapeutic interventions such as hypertonic saline [6]. It has been postulated that hyperchloremia may worsen neurologic outcomes and overall mortality among critically ill TBI patients [7]. From a neurological perspective, there are several mechanisms that may explain the association between chloride and worse outcomes. Animal data demonstrates that hyperchloremia results in a paradoxical reversal in GABA-mediated chloride channel activity [8,9]. Under normal conditions, GABA induces chloride influx due to a low intracellular [Cl^−^] leading to membrane hyperpolarization. However, in the setting of hyperchloremia, GABA induces Cl^−^ efflux due to a high intracellular [Cl^−^] causing membrane depolarization. This mechanism has been postulated to be the reason why patients with hyperchloremia suffer from a higher seizure burden [5,9,10]. Moreover, increasing intracellular [Cl^−^] has been shown to be a major mechanistic driver of cytotoxic oedema [11]. Increased CNS [Cl^−^] and subsequent Cl^−^ influx is an essential step in the development of cerebral oedema, with chloride influx accompanied by corresponding water movement intracellularly and subsequent increased intracellular volume [12,13].

In the context of TBI, the incidence of hyperchloremia may be related at least partially to the use of osmotic agents such as hypertonic sodium chloride (NaCl) solutions. The use of these solutions is commonplace as treatment for traumatic intracranial hypertension (tIH) [1], where they induce an osmotic gradient which causes a shift in fluid out of central nervous system (CNS) cells. This in turn causes a rapid reduction in CNS cellular volume resulting in a fall in intracranial pressure (ICP) and preservation or restoration of cerebral perfusion. Administration of hypertonic NaCl for management of tIH is a cornerstone of modern intensive care practice and is recommended in all relevant guidelines [6,14]. The role of chloride administration in this context and the development of hyperchloremia is not well established.

Hyperchloremia has been linked to increased mortality in critically ill patient groups. It was independently associated with increased mortality in major trauma patients and pediatric multitrauma patients (which include some patients with TBI) [15,16]. Hyperchloremia has also been associated with increased mortality in intensive care unit (ICU) patients [17,18,19] and critically ill septic patients [20]. Finally, in an analysis of MIMIC-III data of a cohort of 48,074 general ICU patients, Yeh et al. demonstrated that, after adjusting for confounders, both hyperchloremia and an increased hyperchloremic burden were associated with an increased mortality, risk of multiorgan dysfunction (MODS) and acute kidney injury [18]. It is thus reasonable to consider that hyperchloremia may also be associated with increased mortality, specifically in the TBI population.

Given the potential role of chloride ions in the mediation of secondary injury to the brain following a primary TBI, and the supporting biological and clinical rationale, we have conducted this narrative review to explore the topic of hyperchloremia in patients with TBI. The objectives of this review were to answer the following questions:

1. Is there a correlation between high chloride levels and mortality or poor neurological outcome in patients with TBI?
2. Is there a correlation between high chloride levels and raised ICP?
3. Does the administration of high chloride solutions contribute to burden of hyperchloremia?

## 2. Materials and Methods

The following databases were examined on 25 November 2025 to undertake this literature review: Pubmed, Medline, Embase and Cinahl. The search strategy used the following Medical Subject Headings (MeSH) terms: ‘chloride’, ‘electrolytes’, ‘hyperchloremia’, AND ‘TBI’, ‘TBI’ OR ‘head injury’. All MeSH terms were exploded. For this narrative review, all dates and study types were eligible for inclusion if relevant information was identified, and all age groups and TBI severities were included. Papers were excluded if they were not in the English language. Titles and abstracts were reviewed, papers were retrieved and examined for relevance to the subject. In addition, references of identified papers were searched and relevant articles retrieved.

## 3. Results

### 3.1. Biological Mechanisms

**Figure 1:**
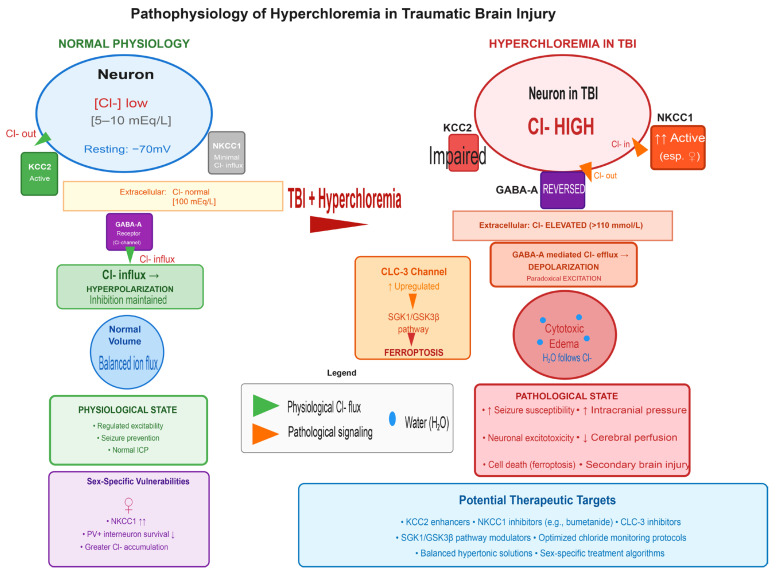
Pathophysiology of hyperchloremia in traumatic brain injury. (**left**) Normal physiology showing KCC2-mediated chloride export, low intracellular chloride, and GABA-mediated hyperpolarization/inhibition. (**right**) Hyperchloremic state after TBI showing KCC2 impairment, NKCC1 upregulation, reversed GABA signaling leading to paradoxical excitation, CLC-3-mediated ferroptosis via SGK1/GSK3β pathway, and cytotoxic edema development. Sex-specific vulnerabilities and potential therapeutic targets are highlighted.

The biological rationale for chloride-mediated neurotoxicity is increasingly well-established through multiple converging mechanisms (Figure 1). At the cellular level, chloride plays a critical role in neuronal excitability through its interaction with gamma-aminobutyric acid receptors, the primary inhibitory neurotransmitter system in the central nervous system [9]. Under normal physiological conditions, GABA receptor activation opens chloride channels, allowing chloride influx into neurons due to the low intracellular chloride concentration maintained by the potassium-chloride cotransporter 2 [11]. This influx hyperpolarizes the neuronal membrane, producing inhibitory effects that are essential for regulating neuronal excitability and preventing seizures [10]. However, in the setting of hyperchloremia, this protective mechanism undergoes a pathological reversal. Elevated extracellular chloride concentrations increase intracellular chloride levels, fundamentally altering the chloride equilibrium potential [9]. When GABA receptors are activated in this altered ionic environment, chloride efflux rather than influx occurs, resulting in membrane depolarization instead of hyperpolarization [5]. This paradoxical excitation transforms an inhibitory neurotransmitter into an excitatory signal, substantially increasing seizure susceptibility and neuronal excitotoxicity [10]. Recent mechanistic evidence has identified chloride channel-3 as a critical regulator of this process in TBI, with studies demonstrating that upregulation of this chloride channel promotes ferroptosis, an iron-dependent form of regulated cell death, through activation of the serum and glucocorticoid-regulated kinase 1 and glycogen synthase kinase 3 beta signalling pathway [21]. Importantly, knockdown of chloride channel-3 in experimental models reduces ferroptosis, alleviates neuronal injury, and improves neurological outcomes, providing proof-of-concept for chloride channel modulation as a therapeutic target [21].

Beyond its effects on neuronal excitability, dysregulated chloride homeostasis contributes directly to the development of cytotoxic edema. Neurons are highly vulnerable to osmotic stress and volume changes [5]. The regulation of neuronal volume depends critically on the balance between inward and outward flux of ions, particularly chloride, through cation-chloride cotransporters and other membrane channels [11]. Increasing intracellular chloride concentration serves as a major mechanistic driver of cytotoxic edema, as the accumulation of intracellular chloride is obligatorily accompanied by water movement into cells to maintain osmotic equilibrium [13]. This process is mediated by dysregulation of the sodium-potassium-2-chloride cotransporter 1 and the potassium-chloride cotransporter 2, which normally maintain the electrochemical gradient for chloride across neuronal membranes [11]. Following TBI, expression and function of these transporters become disrupted, with recent evidence demonstrating sex-specific patterns of dysregulation [22]. Female animals show selective upregulation of sodium-potassium-2-chloride cotransporter 1 following moderate TBI, accompanied by reduced parvalbumin-positive interneuron survival, whereas males exhibit decreased brain-derived neurotrophic factor expression [22]. These findings suggest that chloride dysregulation may contribute to sex-specific vulnerabilities in TBI outcomes and highlight the potential importance of considering biological sex in developing chloride management strategies. The resulting chloride accumulation and cellular swelling not only compromise neuronal function but also contribute to increased intracranial pressure, reduced cerebral perfusion, and propagation of secondary injury cascades that extend well beyond the initial site of trauma [12,13]. Further, in rat models of TBI (and intracerebral hemorrhage) chloride accumulation appears to spread along the white matter tracts and around lateral ventricles, often spreading a considerable distance from the site of injury [23,24].

### 3.2. Incidence of Hyperchloremia

Normal serum chloride levels are generally considered to be 96–106 mEq/L, and high chloride is typically defined as a serum chloride of >110 mEq/L [2,25]. While there are studies reporting on other electrolyte imbalances in the TBI population [26,27,28,29], only three studies [30,31,32] report specifically on the incidence of hyperchloremia, with small patient numbers, and variable results and reporting. A cross-sectional study of 50 patients admitted to a regional trauma center investigated electrolyte disturbances in TBI patients [30]. They reported hyperchloremia was present in 11 of 50 patients (22%) within 24 h of admission. This study reported on all severity types of TBI, with only 7 out of these 11 patients suffering moderate or severe TBI, the remainder were mild. Another observational study of electrolyte disturbances in TBI patients by Raj et al. [31]. reported that 54 of a cohort of 200 TBI patients (27%) developed hyperchloremia during their admission; however, neither a specific timeframe nor the severity of TBI was reported in this single center study. Additionally, neither of these studies reported on the administration of prehospital osmotherapy agents, which may have confounded results. Finally, Roquilly et al. performed a post hoc analysis of a retrospective study in TBI patients receiving isotonic sodium chloride solution as a basal infusion; 65% of the patients experienced hyperchloremia within the first four days before any hypertonic saline infusion was administered [32].

### 3.3. Chloride Load and Levels

Several studies report on the differing chloride levels between patients treated with sodium chloride versus other substances during their osmotherapy intervention for TBI. A randomized controlled trial (RCT) by Roquilly et al. compared isotonic sodium chloride with balanced crystalloid solutions for resuscitation of 42 patients with severe TBI (GCS ≤ 8) or aneurysmal subarachnoid hemorrhage (aSAH) [33]. The total amount of chloride infusion was lower in the balanced group than in the saline group (median 744 mmol and 918 mmol, respectively (*p* = 0.014)). There was a greater proportion of hyperchloremic metabolic acidosis in the sodium chloride group (90% in the saline group vs. 50% of the balanced solutions group, *p* = 0.01)) in the first 48 h. No breakdown was provided for differences between TBI versus aSAH, in this small pilot trial.

Conversely, Bourdeaux and Brown compared hypertonic 5% sodium chloride with hypertonic 8.4% sodium bicarbonate for treatment of 20 episodes of raised intracranial pressure (≥20 mmHg for ≥5 min) in 11 adult patients with severe TBI [34]. Despite the lower chloride load in the sodium bicarbonate group, they reported no significant difference in chloride levels either between groups or compared to their baseline levels. This study was small and underpowered and may not have detected patient-important differences with sodium chloride versus sodium bicarbonate osmotherapy.

Ichai et al. compared 48-h continuous infusions of either sodium lactate or isotonic saline for the prevention of raised ICP in 60 patients with severe TBI [35]. They found that although the cumulative chloride intake was significantly increased in the saline group at 48 h, both treatment groups had a significant increase in serum chloride concentration, with no significant differences noted between the two groups.

A retrospective study of 129 TBI patients treated with hypertonic saline found that while maximum chloride concentration was a strong predictor of acute kidney injury development, the total chloride load administered did not differ between patients who developed acute kidney injury and those who did not [36]. This finding suggests that the peak serum chloride concentration achieved, rather than the cumulative chloride administered, may be the more important determinant of adverse outcome [36]. This study also revealed that non-hypertonic saline sources provided more than 40% of the total chloride load in both groups, highlighting the often-overlooked contribution of medications, maintenance fluids, blood products, and other chloride-containing solutions to overall chloride burden.

Finally, Piper and Harrigan reported on a retrospective cohort study of 32 pediatric patients admitted to ICU with TBI requiring ICP monitoring, and who were treated with hypertonic saline for ICP management [37]. They reported no correlation between hypertonic saline load and peak chloride concentrations (R^2^ = 0.09). There was also no association between hypertonic saline load and metabolic acidosis in this study.

### 3.4. Intracranial Pressure

We did not identify any studies which reported directly on the impact of hyperchloremia on ICP in TBI patients. In the previously mentioned RCT by Roquilly et al. [33], they reported higher serum chloride levels in the saline group (mean difference = 4.8 mmol/L (1.9 to 7.6); *p* = 0.002), however there was no difference in ICPs between groups. Conversely, the RCT by Ichai et al. comparing sodium lactate with sodium chloride infusions demonstrated 50% less occurrence of raised ICP episodes in the sodium lactate group [35]. Importantly, neither of these studies directly compared chloride levels with ICP.

The only other information available on this topic comes indirectly from a machine learning study which examined the use of artificial neural network algorithms to predict deterioration in cerebral perfusion pressure. In analysis of predictive factors used by the algorithms, venous chloride, along with systolic blood pressure coefficient of variation, PaCO_2_, prothrombin time, cerebral perfusion pressure (CPP) coefficient of variation, and mean CPP were found to be most predictive of a deterioration in CPP [38]. While mechanistically plausible [5,12,13], the potential link between hyperchloremia, cerebral oedema and intracranial hypertension has yet to be explored. 

### 3.5. Mortality and Neurological Outcomes

Qureshi et al. performed a post-hoc analysis of the Resuscitation Outcomes Consortium Hypertonic Saline (ROC HS)-TBI study data, and analyzed data from 991 patients with severe TBI [7]. Patients with hyperchloremia in the first 24 h of admission were more likely to require IV mannitol and undergo ventriculostomy or craniotomy during the first five days of admission. Further, patients with greater than or equal to two occurrences of hyperchloremia, compared to those with no hyperchloremia, had significantly higher odds of death within 180 days (OR, 2.35; 95%CI, 1.21–4.61). This association was independent of other known predictors of outcome including age, admission GCS score, CT scan classification (Marshall grades), and ISS. There was a significant association between AUC of serum chloride concentrations and death within 180 days (OR, 0.999; 95% CI, 0.999–1.000; *p* = 0.026). However there was no association between AUC of serum chloride concentrations and death, vegetative state, or severe disability at 180 days (OR, 1.000; 95% CI, 0.999–1.000; *p* = 0.996). There was no association between baseline hyperchloremia and death within 180 days (OR, 0.998; 95% CI, 0.97–1.03; *p* = 0.872) and death, vegetative state, or severe disability at 180 days (OR, 0.98; 95% CI, 0.95–1.01; *p* = 0.113). Cause of death was not reported on.

Consistent with these findings, Ditch et al. found that, after controlling for the burden of hypernatremia and hyperchloremia, only hyperchloremia was independently linked with an increase in mortality in a post-hoc analysis of the Outcome Prognostication in TBI (OPTIMISM) study data [39]. This study examined 458 patients with moderate or severe brain injury. Time-weighted-average (TWA) chloride independently predicted in-hospital mortality (per 10 mmol chloride/L change: adjusted OR 2.9 [95% CI 0.51–4.4]).

Săcărescu et al. report a significant negative correlation between chloride level and Glasgow Coma Scale (GCS) scores (ρ = −0.515; *p* = 0.002) in their cross-sectional analysis of 50 TBI patients [30]. They also noted a significant difference in chloride levels between those who did or did not undergo surgical intervention, with non-surgical patients (mean rank = 14.26) having lower chloride levels than surgical patients (mean rank = 21.79) (U = 68.5, *p* = 0.031). They did not comment on any osmolar therapy received by the patients, which may have confounded results.

A prospective observational study of TBI patients in India reported poor outcome (they defined as ‘expired’ or ‘deteriorated’) in 29 out of 54 patients with hyperchloremia [31]. No further breakdown or clarification of these terms was provided, limiting the utility of these results.

### 3.6. Comparative Effectiveness of Balanced Solutions Versus Saline in TBI

The choice between balanced crystalloid solutions and saline for fluid resuscitation in TBI patients has generated conflicting evidence regarding patient outcomes. The BEST-LIVING systematic review and individual patient data Bayesian meta-analysis of 34,685 critically ill patients across six randomized controlled trials, included 1961 patients with TBI [40,41]. In this TBI subgroup, balanced crystalloid use was associated with increased in-hospital mortality compared to saline (19.1% vs. 14.7%, odds ratio 1.424, 95% confidence interval 1.100–1.818), with a probability of 0.975 that balanced solutions increased mortality in TBI patients. This finding contrasts with the overall study population, where balanced solutions showed a high probability of reduced mortality.

Supporting these observations, a secondary analysis of the SMART trial examined 1157 critically injured patients with TBI randomized to receive balanced crystalloids or saline [42]. While 30-day in-hospital mortality was similar between groups (16% vs. 14%, adjusted odds ratio 1.03, 95% confidence interval 0.60–1.75), patients in the balanced crystalloid group were significantly more likely to die or be discharged to another medical facility rather than home (adjusted odds ratio 1.38, 95% confidence interval 1.02–1.86, *p* = 0.04), suggesting worse functional outcomes despite receiving solutions with more physiological electrolyte composition.

However, this pattern is not universal across all studies. A randomized controlled trial comparing normal saline with Plasmalyte-148 in 90 TBI patients undergoing emergency craniotomy found that Plasmalyte-148 maintained a more favorable metabolic profile with significantly higher pH values, lower base excess, and lower chloride levels, while achieving comparable brain relaxation scores and coagulation profiles [43]. Serum creatinine and urinary injury biomarkers were significantly higher in the normal saline group, suggesting potential renal protective effects of balanced solutions. While this trial was conducted in patients with TBI only, it had a far smaller sample size than the aforementioned meta-analysis and secondary analysis [41,42].

A post-hoc analysis of the COBI trial [44], which examined 370 patients with moderate-to-severe TBI receiving continuous infusion of 20% hypertonic saline, provides additional context regarding chloride load. Despite patients in the intervention arm receiving significantly higher amounts of chloride during the first four days (97.3 ± 31.6 g vs. 61.3 ± 38.1 g, *p* < 0.001) and having higher blood chloride levels at day 4 (117.9 ± 10.7 mmol/L vs. 111.6 ± 9 mmol/L, *p* < 0.001), the incidence of acute kidney injury was not statistically different between groups (24.5% vs. 28.9%, *p* = 0.45).

Key literature is summarized in Table 1.

## 4. Discussion

Our review reveals a complex and at times paradoxical relationship between plasma chloride levels and outcomes in TBI patients. Importantly, it should first be noted that inconsistent reporting and heterogenous patient groups among available studies means interpretation of these results should be undertaken with caution.

There are three main findings to emerge from the available literature. First, hyperchloremia is consistently associated with increased mortality in TBI patients [7,39], and this association persists independent of concomitant hypernatremia. Second, despite mechanistic plausibility [5,13], no studies have directly demonstrated a correlation between elevated chloride levels and raised intracranial pressure measurements. Third, the relationship between hypertonic saline administration and hyperchloremia burden is inconsistent [44], and recent large-scale trials suggest that balanced crystalloid solutions, despite having lower chloride content, may lead to worse outcomes in TBI patients compared to saline [41,42]. These findings have important implications for fluid management strategies in neurologically injured patients and highlight significant gaps in our understanding of chloride’s role in secondary brain injury.

The clinical evidence linking hyperchloremia to adverse neurological outcomes in TBI patients is substantial, though questions remain regarding causality versus association. Two retrospective studies independently linked hyperchloremia with mortality [7,39]. In (ROC HS)-TBI, patients experiencing two or more episodes of hyperchloremia had significantly higher odds of death, and the relationship was dose dependent [7]. In a separate post-hoc analysis of the Outcome Prognostication in TBI study, hyperchloremia was independently linked with increased mortality [39]. Time-weighted-average chloride independently predicted in-hospital mortality, demonstrating that the chloride effect is not merely a reflection of concurrent sodium disturbances and cannot be solely attributable to osmotherapy received. However the causes of death were not outlined in these studies, and may further be confounded by decisions regarding prognostication in severe TBI. For example, it is possible that more severe injuries not only receive more intense therapies, but may also lead to earlier withdrawal of life-sustaining measures due to likely poorer prognosis, which may falsely elevate both chloride levels and mortality rates. Cross-sectional analyses have further demonstrated a significant negative correlation between chloride levels and Glasgow Coma Scale scores, and patients requiring surgical intervention had significantly higher chloride levels than those managed conservatively [30]. Importantly, the interpretation of these associations is complicated by the challenge of distinguishing whether hyperchloremia is directly related to these poor outcomes or serves as a marker of illness severity, treatment intensity, and secondary complications.

An intriguing distinction emerges from recent data regarding chloride level versus chloride load. In Briscoe et al.’s study, maximum chloride concentration was a strong predictor of acute kidney injury development, however the total chloride load administered did not differ between patients who developed acute kidney injury and those who did not [36]. This finding suggests that the peak serum chloride concentration achieved, rather than the cumulative chloride administered, may be the more important determinant of adverse outcome [36]. This study also revealed that non-hypertonic saline sources provided more than 40% of the total chloride load in both groups, highlighting the often-overlooked contribution of medications, maintenance fluids, blood products, and other chloride-containing solutions to overall chloride burden. These observations raise important questions about optimal chloride monitoring strategies and whether interventions should focus on limiting peak chloride levels through more gradual administration or enhanced clearance, rather than simply restricting total chloride intake.

Recent findings from studies comparing balanced crystalloids to saline in critically ill patients present compelling contributions to conventional assumptions about optimal fluid management in TBI. Despite the theoretical advantages of balanced solutions in maintaining more physiological chloride levels and acid–base balance, the BEST-Living meta-analysis demonstrated a clinically concerning 42% relative increase in mortality among TBI patients receiving balanced crystalloids compared to saline [41]. The SMART trial TBI subgroup analysis similarly showed worse discharge disposition with balanced solutions, with patients more likely to be discharged to facilities rather than home or to die [42]. These findings stand in stark contrast to the general critical care literature, where balanced solutions have shown trends toward improved outcomes in broader populations of critically ill patients [42]. Several mechanisms may explain this TBI-specific harm. Balanced crystalloid solutions typically have lower tonicity than saline, which could theoretically worsen cerebral edema through increased water movement into the injured brain. Further, the osmolality of balanced solutions vary between 255–294 mOsm/L, and it may be that even balanced crystalloids, with differing tonicities, may have different effects on TBI patients as a result. The lower osmolality of balanced solutions may fail to provide the sustained osmotic gradient necessary to reduce intracranial pressure in patients with compromised blood-brain barrier integrity. Along with lower osmolality, balanced solutions also have lower sodium concentration than saline. It may be that the protective effect of administering more sodium outweighs the benefit from chloride restriction. Additionally, some balanced solutions contain lactate or acetate as alternative anions, which require hepatic metabolism and could theoretically accumulate in critically ill patients with impaired organ function, potentially affecting cerebral metabolism. However, it is important to note that not all studies demonstrate harm with balanced solutions in TBI. A randomized trial comparing normal saline to Plasmalyte-148 in patients undergoing emergency craniotomy found metabolic advantages and potential renal protection with Plasmalyte-148 without compromising brain relaxation, suggesting that the timing, volume, and clinical context of fluid administration may be critical factors [43]. The post-hoc analysis of the COBI trial adds further nuance to this discussion by demonstrating that continuous infusion of highly hypertonic 20% saline, despite delivering massive chloride loads and producing significant hyperchloremia, did not increase the incidence of acute kidney injury [44]. This finding challenges the assumption that high chloride loads directly cause renal injury in TBI patients receiving hypertonic saline for intracranial pressure management. This finding suggests that the kidney can tolerate substantial chloride exposure when delivered in the context of hypertonic therapy for intracranial hypertension management, perhaps due to the beneficial effects of improved cerebral perfusion and reduced secondary brain injury offsetting any direct nephrotoxic effects of chloride.

Our findings align with and extend observations from other acute neurological conditions where hyperchloremia has been implicated in adverse outcomes. In patients with intracerebral hemorrhage, a change in chloride of 5 mmol/L or greater from baseline within the first 72 h of admission was associated with a higher 90-day mortality, and this association remained significant even after adjusting for concomitant rises in sodium [45]. Moderate hyperchloremia, defined as chloride levels of 115 mmol/L or greater, independently predicted in-hospital mortality in intracerebral hemorrhage patients [46]. Similarly, in patients suffering from large hemispheric infarcts, hyperchloremia was strongly associated with both in-hospital mortality and 3-month mortality, with the relationship persisting after multivariable adjustment for potential confounders [47]. These consistent findings across different acute brain injury phenotypes suggest that chloride dysregulation may represent a common final pathway in neurological deterioration, rather than a phenomenon specific to traumatic injury alone. This is also supported by experimental animal models, where elevated chloride levels, and a similar pattern of chloride ‘spread’ through brain tissue, is observed in rat models of TBI and intracerebral hemorrhage [24]. The universality of these associations across diverse neurological insults strengthens the hypothesis that chloride plays a direct pathophysiological role in secondary brain injury, though definitive proof of causality awaits interventional studies targeting chloride management specifically.

The current evidence base is limited by several important methodological constraints that must be acknowledged. First, the majority of studies examining chloride and outcomes in TBI are retrospective observational analyses subject to confounding by indication, where patients with more severe injuries receive more aggressive treatment including higher volumes of chloride-containing fluids. The few randomized controlled trials examining fluid type in TBI were not specifically designed to test chloride-related hypotheses and used varying definitions of hyperchloremia across studies, ranging from 110 mmol/L to 115 mmol/L or greater. Second, no studies have directly correlated real-time chloride measurements with concurrent intracranial pressure monitoring data, leaving the mechanistic link between chloride and intracranial hypertension inferential rather than empirically demonstrated. The single machine learning study that identified venous chloride as one of the predictive factors for cerebral perfusion pressure deterioration provides only indirect evidence of this relationship [38]. Third, the heterogeneity in osmotherapy protocols, baseline illness severity, concurrent treatments, and outcome measurements makes comparison across studies challenging and limits the ability to conduct meaningful meta-analyses. Fourth, most studies report peak or time-weighted chloride values but do not capture the dynamic evolution of chloride levels over time or the temporal relationship between chloride elevation and clinical deterioration. Finally, there is a paucity of data on long-term functional outcomes beyond mortality, with most studies focusing on short-term endpoints that may not fully capture the impact of chloride dysregulation on neurological recovery and quality of life.

Despite these limitations, the accumulated evidence suggests that hyperchloremia in TBI patients warrants clinical attention as a potentially modifiable risk factor for poor outcomes. The consistent association between elevated chloride and increased mortality, independent of sodium levels, combined with robust mechanistic data linking chloride dysregulation to neuronal excitotoxicity and cytotoxic edema [5,21], provides a compelling rationale for further investigation. However, the findings regarding balanced solutions highlight the complexity of translating these observations into clinical practice. The challenge for clinicians lies in balancing the need to avoid hyperchloremia with the risks associated with balanced crystalloid solutions in TBI patients and the established benefits of hypertonic saline therapy for intracranial hypertension management. Current guidelines [1,48,49] recommend hypertonic saline as a cornerstone of traumatic intracranial hypertension treatment, and the available evidence does not support abandoning this practice based on concerns about chloride exposure alone. Indeed, other therapies for tIH such as escalating sedation, neuromuscular blockade, and barbiturate coma have their own potential complications and this should be considered carefully. Instead, our findings suggest the need for more nuanced approaches that incorporate chloride monitoring into treatment algorithms, potentially using chloride thresholds to guide the choice and rate of fluid administration while maintaining adequate osmotherapy for intracranial pressure control.

## 5. Future Research Priorities

The current evidence base reveals that although hyperchloremia is common among TBI patients, there are substantial gaps in our understanding of the role of chloride in TBI pathophysiology and optimal management strategies. Addressing these knowledge deficits will require carefully designed studies employing rigorous methodologies to establish causality, identify therapeutic targets, and guide clinical practice.

The most pressing need is to more accurately define the incidence of hyperchloremia, and its relationship to injury severity, administration of hyperosmolar therapy and patient outcomes.

A critical evidence gap is the lack of studies directly correlating real-time chloride measurements with concurrent intracranial pressure monitoring data. Future research should employ continuous or frequent simultaneous monitoring of serum chloride concentrations and intracranial pressure in patients with severe TBI requiring invasive neuromonitoring. Such studies could establish temporal relationships between chloride elevations and intracranial pressure or cerebral compliance changes, determine whether chloride thresholds exist above which intracranial pressure control becomes compromised, and assess whether chloride reduction interventions can effectively lower intracranial pressure. Advanced neuromonitoring techniques including brain tissue oxygen monitoring, cerebral microdialysis, transcranial doppler ultrasonography, and near-infrared spectroscopy could be incorporated to provide additional mechanistic insights into how chloride affects cerebral metabolism, perfusion, and cellular function at the tissue level. These multimodal monitoring approaches would help elucidate whether chloride exerts its effects primarily through osmotic mechanisms affecting bulk fluid shifts, through cellular mechanisms affecting neuronal function and viability, or through a combination of both mechanisms. Currently, only a single study, using machine learning techniques, provides data on the relationship between chloride and raised intracranial pressure, representing a key area of future research in TBI.

Once these relationships have been established, this would allow for adequately powered, prospective randomized controlled trials specifically designed to test chloride-targeted interventions in TBI patients. Such trials should compare chloride-restrictive versus chloride-liberal fluid and osmotherapy management strategies with clearly defined protocols for fluid selection, chloride monitoring thresholds, and treatment algorithms. Standardized reporting of all sources of chloride, including non-osmotherapy sources, should be highly encouraged in TBI studies. These studies must be sufficiently large to detect clinically meaningful differences in patient-centered outcomes including mortality, long-term functional status, and quality of life. Importantly, trial designs should stratify patients by injury severity, gender, presence of tIH, and need for osmotherapy and other tiered therapies to account for the heterogeneity of TBI populations and treatment requirements. Given recent findings showing potential harm with balanced crystalloids despite their lower chloride content, future studies might investigate novel fluid formulations such as balanced hypertonic solutions that combine the osmotic benefits of hypertonic therapy with more physiological electrolyte composition. The development and testing of such solutions could potentially reconcile the apparent conflict between the need to manage intracranial pressure and the desire to avoid hyperchloremia.

The mechanistic studies identifying chloride channel-3 as a critical regulator of ferroptosis in TBI and demonstrating sex-specific dysregulation of sodium-potassium-2-chloride cotransporter 1 and potassium-chloride cotransporter 2 open important avenues for therapeutic development. Future research should investigate whether pharmacological modulation of these chloride transporters and channels can improve outcomes in experimental TBI models and whether such interventions are safe and effective in human patients. The cation-chloride cotransporter inhibitor bumetanide, which blocks sodium-potassium-2-chloride cotransporter 1, has shown neuroprotective effects in neurological conditions in animal models [50] and could be evaluated specifically in TBI populations with hyperchloremia. Similarly, inhibitors of chloride channel-3 or modulators of the serum and glucocorticoid-regulated kinase 1 and glycogen synthase kinase 3 beta pathway might represent novel therapeutic strategies targeting the ferroptosis cascade. Given the sex-specific differences in chloride transporter expression following TBI, future studies should examine whether men and women respond differently to chloride management strategies and whether sex-specific treatment algorithms should be developed. The biological mechanisms underlying these sex differences, including potential roles of hormonal influences on ion transporter expression and function, warrant detailed investigation.

An important unresolved question is the identification of optimal chloride thresholds and targets for TBI management. Current definitions of hyperchloremia vary across studies, and it remains unclear whether these arbitrary cutoffs reflect true pathophysiological inflection points or merely represent convenient statistical divisions. Future research could employ sophisticated analytical approaches such as machine learning techniques to identify chloride levels associated with inflection points in outcome trajectories. These analyses should account for the temporal dynamics of chloride changes, as the impact of sustained hyperchloremia may differ from that of transient elevations. Studies should also determine whether the rate of chloride increase influences outcomes independently of the absolute level achieved, analogous to the established relationship between rate of sodium correction and osmotic demyelination syndrome. Furthermore, research is needed to establish whether chloride targets should be adjusted based on individual patient characteristics such as baseline renal function, presence of metabolic acidosis, severity of neurological injury, and concurrent osmotherapy requirements.

The distinction between chloride level and chloride load as determinants of adverse outcomes requires further clarification through prospectively designed studies that systematically track both parameters. Future research should quantify the relative contributions of different sources to total chloride burden, including hypertonic saline therapy, maintenance fluids, resuscitation fluids, blood products, and medications containing chloride. Such comprehensive chloride accounting could identify opportunities for chloride reduction that do not compromise essential therapies such as osmotherapy for intracranial pressure management. Studies should also investigate whether interventions aimed at enhancing chloride clearance, such as adjustment of renal replacement therapy parameters in patients requiring such support, can safely reduce chloride levels without inducing other electrolyte disturbances or compromising hemodynamic stability. The time course over which hyperchloremia develops and resolves, and the reversibility of chloride-associated injury, remain poorly characterized and warrant systematic investigation.

Long-term functional outcomes beyond mortality represent an important but understudied domain in chloride research. Most existing studies focus on in-hospital or 30-day mortality, but the impact of chloride dysregulation on neurological recovery, cognitive function, return to work, and quality of life at six months, one year, and beyond has not been adequately characterized. Future studies should incorporate validated functional outcome measures such as the Glasgow Outcome Scale—Extended, neuropsychological testing batteries, and quality of life assessments to provide a more comprehensive picture of chloride’s long-term impact on TBI recovery. Longitudinal studies following patients over extended periods could determine whether early hyperchloremia predicts long-term disability independently of initial injury severity and whether the effects of chloride on outcomes are mediated through specific complications such as seizures, infections, or an increased intracranial pressure burden.

Finally, if chloride is clearly demonstrated to be harmful in well-conducted, prospective clinical trials, implementation science research will be needed to translate emerging evidence into clinical practice. Development of clinical decision support tools that integrate chloride monitoring with other physiological parameters to guide fluid management decisions could help clinicians navigate the complex trade-offs between different therapeutic goals. Quality improvement initiatives examining the feasibility and impact of chloride monitoring protocols in real-world settings would provide valuable information about practical barriers to implementation and strategies for overcoming them. Comparative effectiveness research examining different chloride management approaches across diverse practice settings could identify best practices and inform guideline development. Economic analyses evaluating the costs and benefits of intensive chloride monitoring and targeted interventions would help health systems make informed resource allocation decisions. These translational research efforts are essential to ensure that advances in our understanding of chloride’s role in TBI pathophysiology ultimately improve outcomes for patients.

## 6. Conclusions

Hyperchloremia frequently occurs in patients with TBI and appears to be independently associated with increased mortality, although the mechanistic link through which harm may occur remains unestablished through direct correlation studies, and the degree to which chloride serves as a biomarker for more severe injury or directly causes harm is not clear. Recent evidence from large randomized trials suggests that balanced crystalloid solutions, despite having lower chloride content, may be associated with worse outcomes in TBI patients compared to saline, adding further nuance to conventional assumptions about optimal fluid management. The study of hyperchloremia incidence and its main contributing factors relies predominantly on small retrospective studies with variable reporting. Emerging mechanistic data linking chloride channel dysregulation to ferroptosis and cytotoxic edema, along with recognition of sex-specific vulnerabilities in chloride homeostasis, provide compelling biological rationale for chloride’s role in secondary brain injury. However, the clinical findings regarding fluid choice, the absence of ICP–chloride correlation studies, and the distinction between chloride level versus load as determinants of outcomes highlight substantial gaps in our current understanding. Comprehending chloride trends and their possible correlations with clinical outcomes in TBI patients, while navigating the complex balance between avoiding hyperchloremia and maintaining adequate osmotherapy for intracranial pressure management, represents an important area for future investigation and may ultimately inform more refined and personalized fluid, electrolyte, and osmotherapeutic management strategies.

## Figures and Tables

**Table 1:** Studies evaluating hyperchloremia and chloride-rich/balanced fluids in traumatic brain injury (TBI).

Author	Year	Study Design	Sample Size	Patient Characteristics	Data Collected/ Exposure Variables	Key Findings	Notes/Limitations
Bordeaux & Brown [11]	2011	RCT (5% NaCl vs. 8.4% sodium bicarbonate) for intracranial hypertension episodes	11 patients; 20 episodes elevated ICP	Adult patients with severe TBI	Chloride levels during treatment episodes	No significant differences in chloride levels between groups or compared with baseline	Very small study; physiologic endpoint
Roquilly [32]	2011	Single-center retrospective observational (surgical ICU)	50	Severe TBI (GCS < 9) with ICP monitor; continuous hypertonic saline targeting natremia; refractory ICP > 20 mmHg	Serial serum chloride over 24 h	Chloride increased from median 111 (IQR 107–119) at 0 h to 121 (IQR 117–124) at 24 h (*p* < 0.05)	No explicit hyperchloremia threshold stated; reflects HTS strategy
Ichai [35]	2013	Double-blind RCT (2 ICUs): sodium lactate vs. isotonic saline for prevention of raised ICP	60	Adults 18–65 with severe TBI (GCS < 9). Exclusions: pregnancy, cardiac arrest, CSF leak, multiorgan involvement, hyperosmolar agents in prior 6 h	Cumulative chloride intake; serum chloride; neurological outcomes	Both treatments increased chloride; no between-group difference in serum chloride or neurological outcomes. Chloride intake was lower with sodium lactate at 48 h	
Roquilly [33]	2013	Single-center double-blind pilot RCT: balanced isotonic vs. 0.9% saline	42	Severe TBI; expanded to include subarachnoid haemorrhage. Exclusions: multiple trauma, pregnancy, severe metabolic disturbance, hypertonic saline prior to inclusion	Chloride infusion (mmol), serum chloride, osmolality, acid–base; ICP	Balanced group received less chloride and had less hyperchloremic acidosis (90% saline vs. 50% balanced); higher chloride/osmolality with saline; no difference in ICP	Pilot size; not powered for clinical outcomes
Piper & Harrigan [37]	2015	Retrospective cohort (pediatric)	32	Pediatric TBI requiring ICP monitoring	Peak chloride; hypertonic saline load; metabolic acidosis	Peak chloride did not correlate with hypertonic saline load; no association between hypertonic saline load and metabolic acidosis	Small cohort; threshold not defined
Raj [31]	2020	Prospective cross-sectional	200	TBI without polytrauma (ED or outpatient). Exclusions: diabetes, hypertension, thyroid disorders	Electrolytes; CRP at admission, 24 h, days 4/8/12/16	Hyperchloremia: 54/200; reported worse outcomes vs. normochloremia (*p* < 0.05 reported, calculations not shown)	Outcome definitions unclear; no TBI severity breakdown; limited demographics
Ditch [39]	2021	Retrospective observational (OPTIMISM study)	458	Moderate–severe TBI	Time-weighted average (TWA) chloride and sodium; mortality	TWA chloride independently predicted in-hospital mortality; chloride remained significant when modelled with TWA sodium	Hyperchloremia threshold not specified; observational confounding possible
Lombardo [42]	2022	Secondary analysis of SMART trial (balanced crystalloids vs. saline)	1157 (TBI subgroup)	Adult TBI patients admitted to ICU	Incidence of Cl > 110; 30-day in-hospital mortality; discharge disposition	Balanced group less likely to have Cl > 110 (34% vs. 41%; *p* = 0.014). Mortality similar (16% vs. 14%). Balanced group more likely to die or be discharged to another facility rather than home (aOR 1.38; *p* = 0.04)	Subgroup/secondary analysis; discharge disposition composite may be difficult to interpret
Qureshi [7]	2022	Retrospective subgroup analysis (ROC HS–TBI)	991	Severe TBI (GCS < 9). Exclusions: cardiac arrest, drowning, hanging, missing values	Frequency of hyperchloremia occurrences in first 24 h; GOSE at 6 months; 180-day mortality; fluid volume	≥2 hyperchloremia occurrences in first 24 h associated with worse GOSE distribution and higher mortality vs. none; association independent of age, admission GCS, CT classification, and ISS; hyperchloremia group received higher total fluid volume	Residual confounding by fluid volume/illness severity likely
Huet [44]	2023	Post-hoc analysis (COBI trial)—subgroup analysis	322 (TBI subgroup)	Adults 18–80 with moderate–severe TBI (GCS < 13)	Cumulative chloride load (ICU admission to day 4); hyperchloremia exposure; AKI	Intervention had higher cumulative chloride load vs. control (*p* < 0.001); no association between hyperchloremia exposure and AKI	Threshold not defined; post-hoc subgroup
Săcărescu & Turliuc [30]	2024	Cross-sectional observational (first 24 h)	50	All TBI severities. Exclusions: endocrine disorders, paediatric, pregnancy, severe anaemia	Electrolytes; demographics; mechanism; GCS; imaging; surgery	Hyperchloremia 22% (11/50). Higher chloride associated with lower GCS and higher surgical requirement	Osmotherapy exposure not reported (important confounder)
Briscoe [36]	2024	Retrospective observational (AKI risk factors)	129	TBI, age ≥ 12 years, ICU length of stay ≥ 72 h, and administration of ≥24 h of continuous HTS or 500 mL of HTS boluses.	Maximum and change in serum chloride; total chloride load; sources of chloride; AKI	Hyperchloremia (Cl ≥ 115 mEq/L) was more common in the AKI group (100% vs. 81%, *p* = 0.0428); maximum and delta chloride higher in AKI; total chloride load not different; non-hypertonic-saline sources provided >40% of chloride load	Patient details and AKI definition not captured in source table
Sakkanan [43]	2024	RCT: 0.9% saline vs. Plasma-Lyte (perioperative)	90	Adults 18–60 undergoing craniotomy/evacuation of subdural haematoma. Exclusions: multitrauma, severe renal dysfunction, severe electrolyte disturbance, pregnancy, coagulopathy	Acid–base variables; chloride; brain relaxation; coagulation	Plasma-Lyte group had lower chloride and more favourable acid–base profile; comparable brain relaxation and coagulation	Perioperative population; physiologic endpoints
Zampieri [41]	2024	Systematic review and individual patient data meta-analysis (TBI subset)	1961 (TBI subset)	TBI patients within pooled dataset	Balanced crystalloids vs. saline; in-hospital mortality	Balanced crystalloids associated with higher in-hospital mortality vs. saline in TBI subset (reported OR ~1.42)	Subset analysis and heterogeneity considerations; hyperchloremia not primary exposure

Abbreviations: AKI = acute kidney injury; aOR = adjusted odds ratio; Cl = chloride; CRP = C-reactive protein; CSF = cerebrospinal fluid; CT = computed tomography; ED = emergency department; GCS = Glasgow Coma Scale; GOSE = Glasgow Outcome Scale—Extended; HTS = hypertonic saline; ICP = intracranial pressure; ICU = intensive care unit; IQR = interquartile range; ISS = Injury Severity Score; OR = odds ratio; RCT = randomised controlled trial; TBI = traumatic brain injury; TWA = time-weighted average.

## Data Availability

No new data were created or analyzed in this study.

## Abbreviations

The following abbreviations are used in this manuscript:

TBI	Traumatic Brain Injury
ICP	Intracranial Pressure
ICU	Intensive Care Unit
tIH	Traumatic intracranial hypertension
CNS	Central Nervous System
